# Case report: Imaging features of aorta-right atrial tunnel in a dog using two-dimensional echocardiography and computed tomography

**DOI:** 10.3389/fvets.2023.1160390

**Published:** 2023-07-03

**Authors:** Geunha Kim, Yewon Ji, Ho-Gyun Jeong, Taekwon Lee, Kichang Lee, Hakyoung Yoon

**Affiliations:** ^1^Department of Veterinary Medical Imaging, College of Veterinary Medicine, Jeonbuk National University, Iksan-si, Republic of Korea; ^2^Animal Medical Center, Jeonju, Republic of Korea

**Keywords:** canine, aorta-RA tunnel, aorta-RA shunt, right atrium, aortic root, Valsalva, right coronary sinus, cardiovascular anomaly

## Abstract

A 7-year-old castrated male Pomeranian dog weighing 5 kg presented with a right-sided continuous murmur without any clinical signs. Thoracic radiographs indicated cardiomegaly and right atrial (RA) bulging. Echocardiography revealed a tunnel originating from the right coronary sinus of Valsalva and terminating in the RA. Contrast echocardiography revealed pulmonary arteriovenous anastomoses. Computed tomography (CT) demonstrated a tortuous shunting vessel that originated from the aorta extending in a ventral direction, ran along the right ventricular wall, and was inserted into the RA. Based on these diagnostic findings, the dog was diagnosed with the aorta-RA tunnel. At the 1-year follow-up visit without treatment, the dog showed no significant change except for mild left ventricular volume overload and mildly decreased contractility. To the best of our knowledge, this is the first case report of an aorta-RA tunnel that has been described in detail using echocardiography and CT in a dog. In conclusion, the aorta-RA tunnel should be included in the clinical differential diagnoses if a right-sided continuous murmur is heard or shunt flow originating from the aortic root is identified.

## Introduction

A cardiac shunt is a congenital heart defect in which blood flows directly from one side of the cardiac circulation to the other through an abnormal connection (1, 2). In dogs, patent ductus arteriosus and ventricular septal defects are most commonly reported (3–5), while atrial septal, atrioventricular defects, and aortopulmonary window are relatively rare (6–9). However, to our knowledge, aorta-right atrial (RA) tunnel has never been reported in veterinary medicine.

In human medicine, the aorta-RA tunnel was first described in 1980 (10) and has been multiple times reported since then (10–15). Aorta-RA tunnel is defined as a congenital anomaly with a tunnel-like extracardiac communication originating from one of the sinuses of Valsalva (aortic root) and terminating in the RA or the superior vena cava (13, 14), and clinical presentations range from asymptomatic to congestive heart failure (14, 15). The general methods for diagnosing aorta-RA tunnels in human medicine include ascending aortography, coronary angiography, two-dimensional echocardiography, computed tomography, and computed tomographic angiography (10–15).

In our study, we were presented with the first aorta-RA tunnel in a dog and diagnosed the disease using two-dimensional echocardiography and computed tomography (CT). This report aims to describe in detail the echocardiographic and CT imaging features of aorta-RA tunnel in a dog.

### Case description

A 7-year-old castrated male Pomeranian dog weighing 5 kg was presented at the Jeonbuk Animal Medical Center for cardiac screening. The dog had no history of disease or clinical signs associated with the cardiovascular system. On physical examination, the dog had normal vitals (systolic blood pressure, 135 mmHg; heart rate, 120 beats; and temperature, 38.3°C) but a right-sided continuous murmur was noted. Blood tests showed no abnormal findings. Thoracic radiography (HF-525 PLUS, ECORAY, Seoul, Korea) revealed an increased vertebral heart score (12.6; reference range, 8.7–10.7), and sternal contact (4; reference range, 2.5–3.0) on the lateral view. Generalized cardiomegaly and a bulge at 9–11 o’clock of the cardiac silhouette were identified on the ventrodorsal view. The diameter of pulmonary vessels and the caudal vena cava were within the normal reference range.

**Figure 1:**
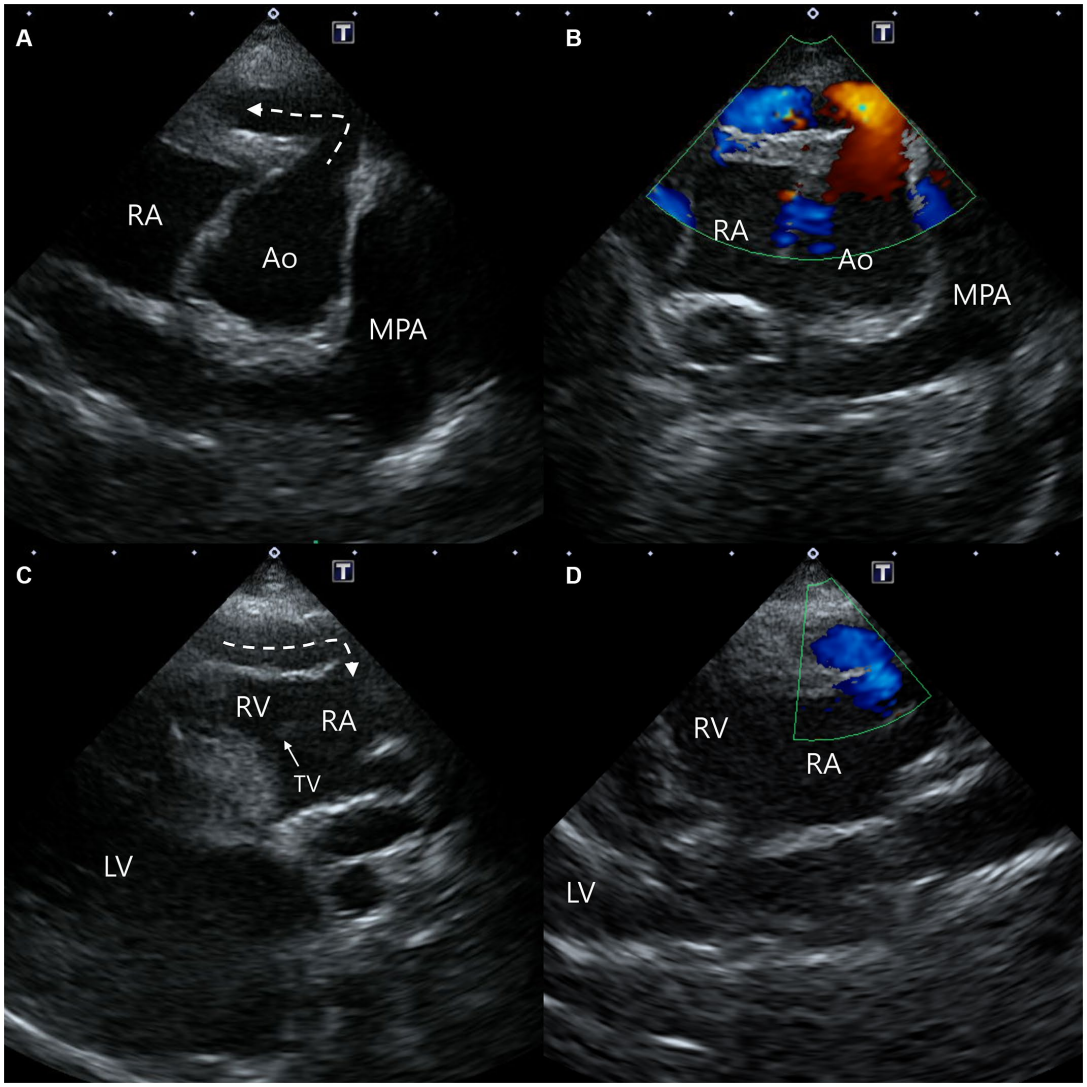
Two-dimensional and color Doppler echocardiogram images of the origin **(A,B)** and termination **(C,D)** of the aorta-right atrial tunnel. The tunnel originated from the right coronary sinus of Valsalva in the ventral direction and then ran rightward on the parasternal short-axis view **(A,B)**. The tunnel running adjacent to the right ventricular and right atrial free wall was terminated in the right atrium from the modified right parasternal long-axis view **(C,D)**. Ao, aorta; LV, left ventricle; MPA, main pulmonary artery; RA, right atrium; RV, right ventricle; TV, tricuspid valve.

All two-dimensional echocardiographic examination was performed using a 5-MHz phased array transducer (Aplio 300; Canon Medical System, Europe B.V., Zoetermeer, Netherlands). On echocardiography, the aorta-RA tunnel was identified. The tunnel originated from the right coronary sinus of Valsalva extending in a ventral direction and the diameter of the origin orifice was measured to be about 6 mm from the right parasternal short-axis (level of aortic root) view (Figures 1A,B). The tunnel running adjacent to the right ventricular (RV) and RA-free wall was tortuous, and was observed from the right parasternal long-axis five-chamber view, terminating in the RA from the modified right parasternal long-axis view. The termination orifice diameter was approximately 2.3 mm, much smaller than the origin orifice diameter (Figures 1C,D). Color Doppler echocardiography demonstrated blood flow from the aorta to the RA (Figures 1B,D), and continuous-wave Doppler profile obtained across the shunt site showed a continuous wave with systolic and diastolic pattern with a pressure gradient of 7.8 mm Hg.

**Figure 2:**
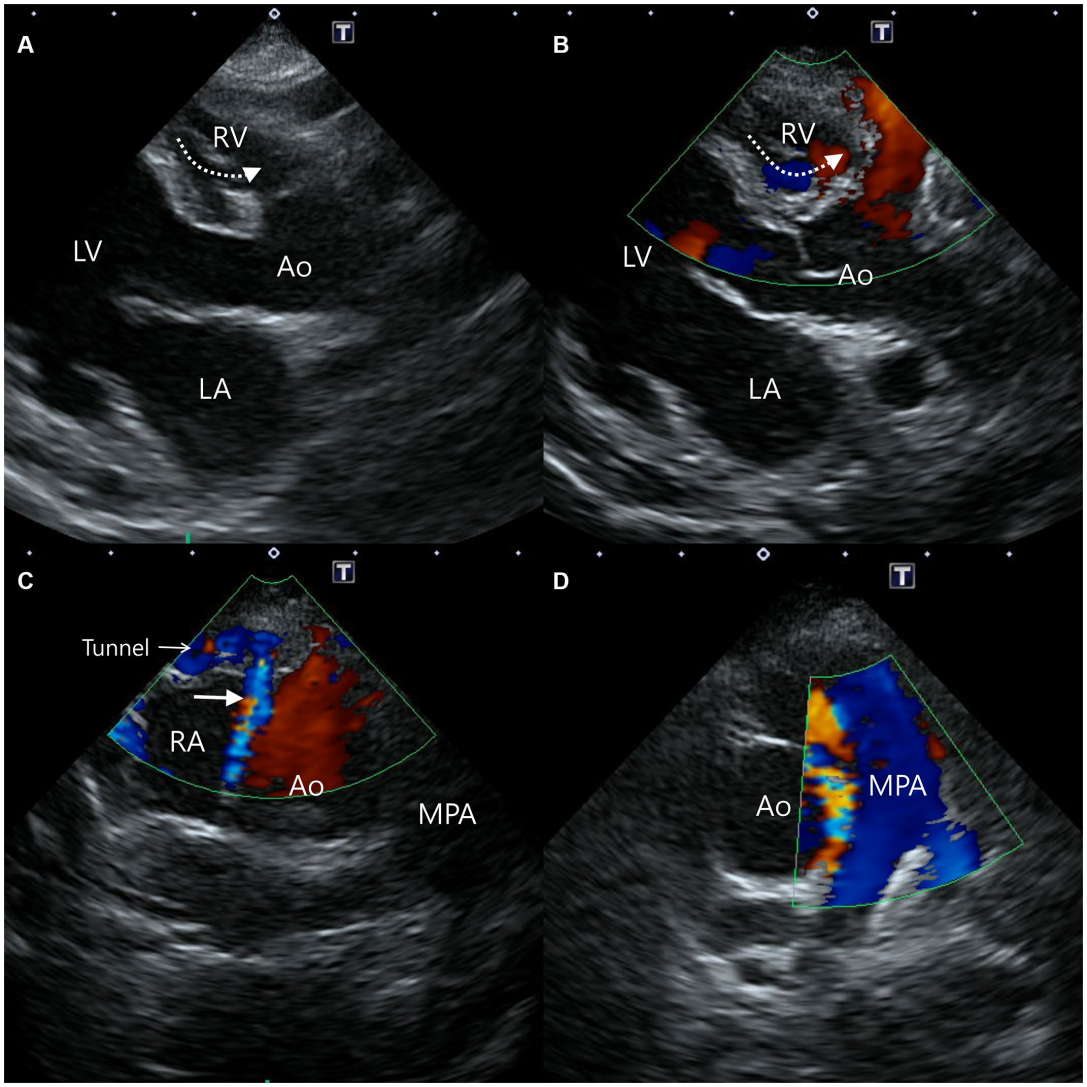
Two-dimensional and color Doppler echocardiogram images of concurrent findings. Blood flow from the vessel (dotted arrow) within the interventricular septum to the right ventricle was confirmed from a modified right parasternal long-axis view **(A,B)**. From the right parasternal short-axis view, blood flow (solid arrow) from the proximal aorta-right atrial tunnel runs between the aorta and the right atrium **(C)**, and the mosaic pattern between the aorta and the main pulmonary artery **(D)** were identified. Ao, aorta; LA, left atrium; LV, left ventricle; MPA, main pulmonary artery; RA, right atrium; RV, right ventricle.

In addition, a small communication between the blood vessel within the interventricular septum and the RV was confirmed (Figures 2A,B). Moreover, another blood flow arising from the proximal aorta-RA tunnel was also identified, and it runs between the aorta and the RA from the right parasternal short-axis (level of the aortic root) view, which was considered the dilated RCA branch (Figure 2C). On the same view, since the mosaic pattern was observed between the ascending aorta and the main pulmonary artery, the aortopulmonary window and a supracristal ventricular septal defect were included in the differential diagnoses (Figure 2D) (9, 16).

Concurrent findings include mild LV enlargement and mildly decreased contractility: LA:Ao = 1.21; LVIDDN = 2.00; LVIDSN = 1.47; EDVI = 139.00; ESVI = 70.76; FS = 23.99; EF = 51.1. LV volume parameters were calculated by the Teichholz method. In addition, the following parameters which indicate mildly impaired relaxation were acquired: E:A = 0.82; E:E’ = 12.39; E’:A’ = 0.69; Tei index = 0.64; E peak = 66.9; E:IVRT = 0.97. The left atrium, right atrium, and right ventricle were normal in size and no mitral, tricuspid, aortic, or pulmonic valve regurgitation was identified. Therefore, the cardiomegaly on the radiography was considered to be the summation of the aorta-RA tunnel and the heart silhouette, not due to actual atrial or ventricular dilations.

Contrast echocardiographic studies were performed using a left apical long-axis four-chamber and an intravenous hand-agitated saline injection. An intravenous catheter was placed into the cephalic vein and attached with a three-way stopcock. Two 10 mL syringes were fixed to the three-way stopcock and one syringe was filled with 8 mL hand-agitated saline. The 2 mL of saline contrast bubbles were injected into the cephalic vein by forced hand injection. This process has been performed four times in the same way. The microbubbles were identified in LV five to six cardiac cycles after RV opacification and were not seen in the abdominal aorta. Intracardiac shunts or right-to-left shunts were not observed, and intrapulmonary arteriovenous anastomoses were demonstrated using saline contrast echocardiography (17–19).

**Figure 3:**
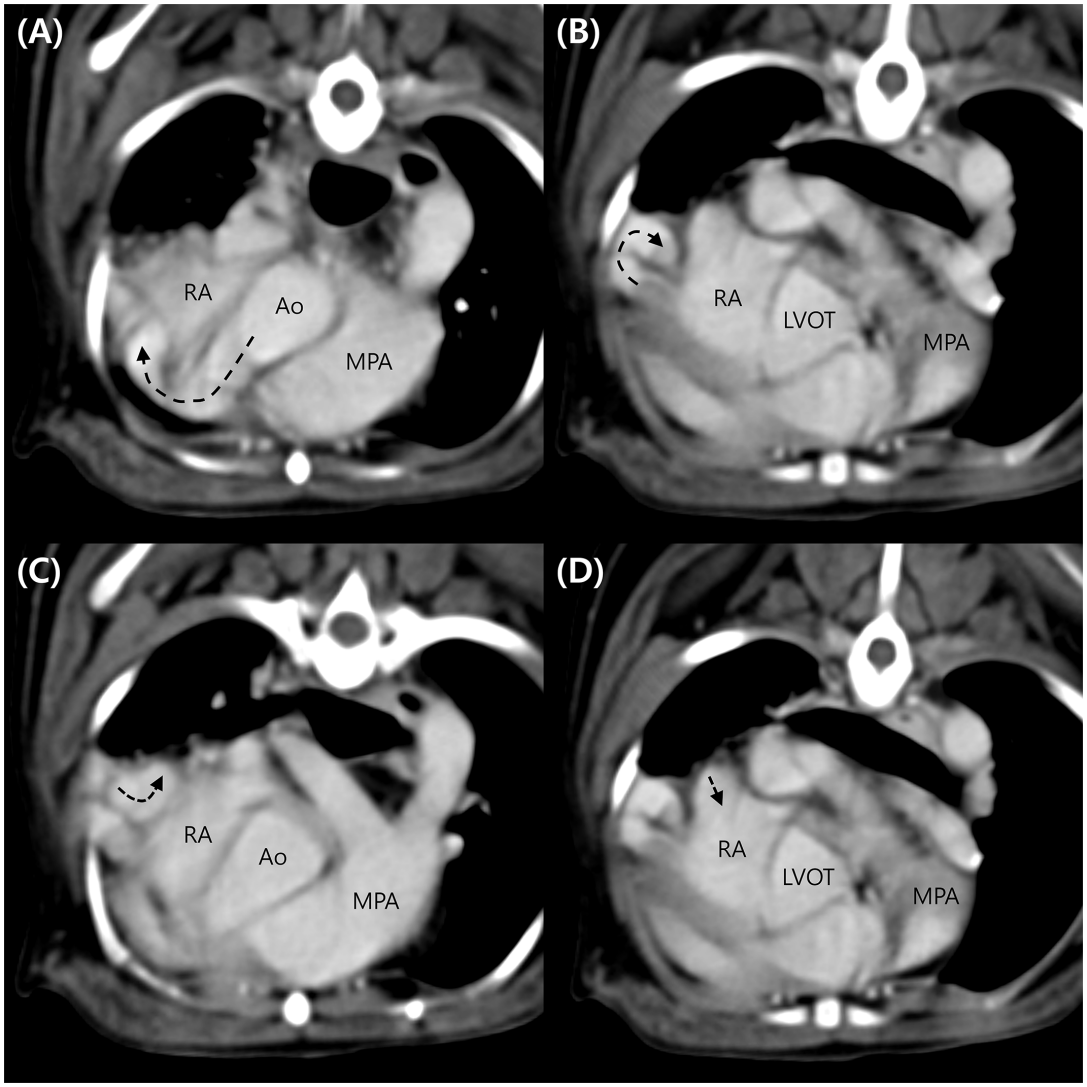
Computed tomography images of the aorta-right atrial tunnel. The tunnel originating from the right coronary sinus of Valsalva extending in a ventral ventral direction runs rightward **(A)**, caudodorsal to the aortic root **(B)**. After a tortuous course adjacent to the right ventricular and right atrial free wall **(C)**, the tunnel entered the right atrium **(D)**. Ao, aorta; LVOT, left ventricular outflow tract; MPA, main pulmonary artery; RA, right atrium.

**Figure 4:**
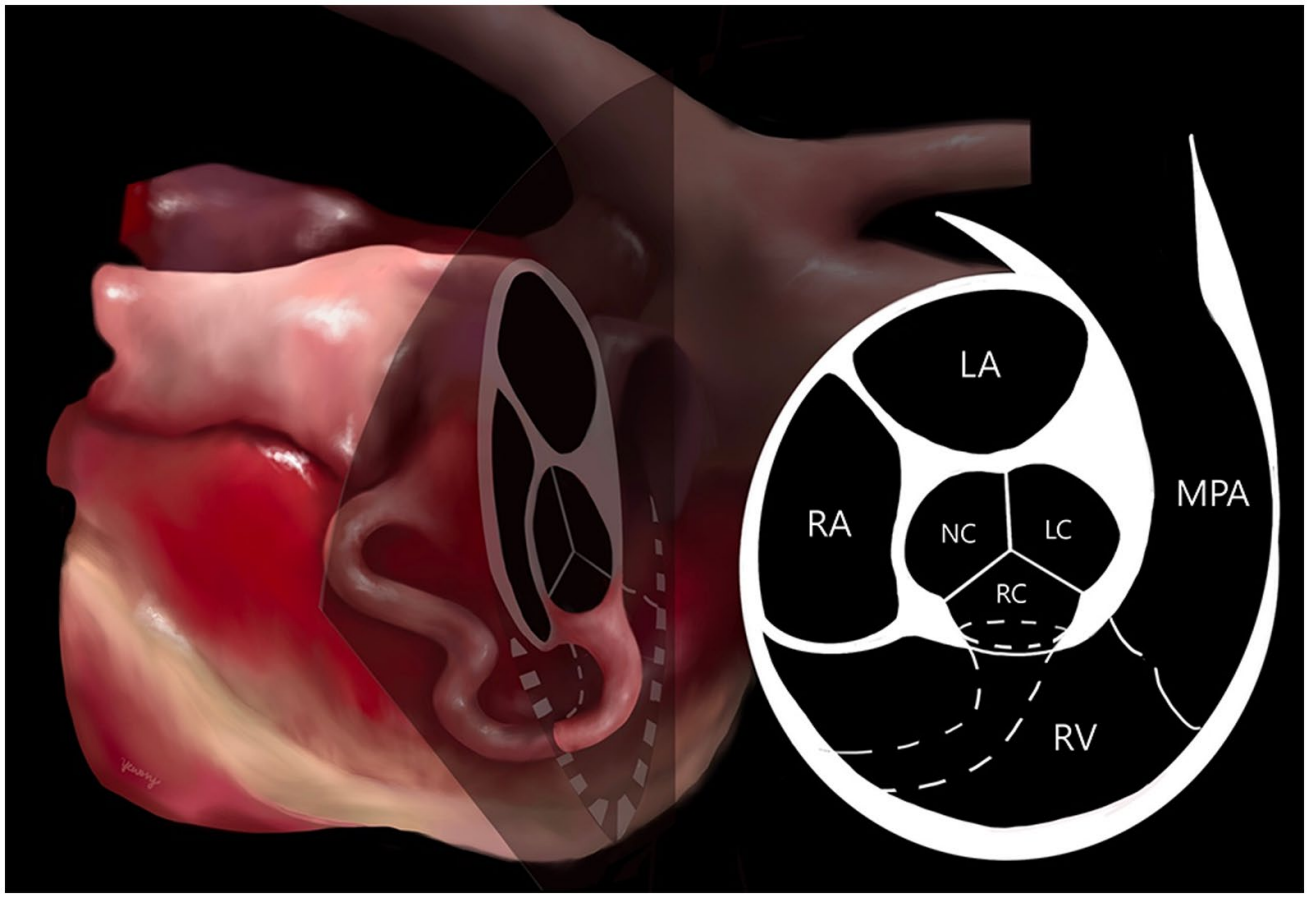
Illustration of the aorta-right atrial tunnel made with reference to the three-dimensional computed tomographic reconstruction. The origin orifice can be observed on the cross-section of the heart at the aortic valve level. The tunnel’s origin from the right coronary sinus of Valsalva and entry to the right atrium through a tortuous communication was confirmed. LA, left atrium; LC, left coronary cusp; MPA, main pulmonary artery; NC, non-coronary cusp; RA, right atrium; RC, right coronary cusp; RV, right ventricle.

Computed tomography (Alexion, TSX-034A, Toshiba Medical Systems, Tochigi, Japan) examination was performed to delineate the extracardiac anatomy of the aorta-RA tunnel. Non-gated CT scanning was conducted under general anesthesia with induction using 0.2 mg/kg butorphanol (Butophan; Myungmoon Pharm Co., Ltd., Seoul, Korea) and 6 mg/kg propofol (MCT/LCT 1%, Freefol-MCT; Daewon Pharm Co., Ltd., Seoul, Korea) and maintenance with 1.5% isoflurane (Isoflurane; Hana Pharm Co., Ltd., Hwaseong, Korea). The CT scanning parameters were 100 kVp, 150 mA, 256 × 256 matrix, 240 mm FOV, 3 mm collimation thickness, and pitch of 1.0. Breath-holding for motion pause was achieved after hyperventilation and positive end-expiratory pressure with 15 cmH2O during scanning. Pre-contrast and post-contrast CT images were acquired. Nine hundred milligrams iodine/kg of Iohexol contrast medium (Omnipaque, GE healthcare, United States) was intravenously injected at the injection rate of 3 mL/s with a power injector and the post-contrast scan was performed 60 s after the first injection. CT images revealed a tunnel originating from the right coronary sinus of Valsalva extending in a ventral direction, running rightward, caudal to the aortic root along the RV and RA-free wall, and terminating in the RA (Figure 3). An illustration made with reference to the three-dimensional CT reconstruction helped delineate the morphologic structure of the aorta-RA tunnel (Figure 4).

On follow-up at 1 year, the dog was still asymptomatic, and thoracic radiography revealed no significant change. Compared to the previous year, echocardiography confirmed mild LV volume overload and mildly decreased contractility: LA:Ao = 1.2; LVIDDN = 2.15; LVIDSN = 1.64; EDVI = 168.18; ESVI = 94.04; FS = 21.25; EF = 44.08. No significant change was identified in other indicators. The pulmonary flow (Qp) was assessed in the pulmonary artery and the systemic flow (Qs) was assessed in the aorta after the aorta-RA tunnel. Qp/Qs ratio was estimated at 1.3 (reference range 0.71–1.29) (20).

## Discussion

Aorta-RA tunnel is defined as an abnormal tubular extracardiac communication between the aortic root and the RA or the superior vena cava in human medicine (14, 15). Several hypotheses on the cause have been suggested. A more likely cause seems to be a congenital deficiency of the elastic lamina in the aortic media that gradually enlarges due to elevated aortic pressure to form an extracardiac tunnel (14, 15). Alternatively, aneurysmal dilation of the sinus nodal artery has been proposed as the embryologic basis of this lesion. (11, 14). Other hypotheses include an abnormal supravalvular ridge formation and mesocardial cyst persistence (10).

Aorta-RA tunnel has been reported sporadically in human medicine (10–15). However, it has not been previously reported in veterinary medicine to our knowledge. One similar case report for a dog with an RCA to RA tunnel also featured communication between the aorta and the RA, but it is clearly different in that the origin of the fistula was the right coronary artery, not one of the sinuses of Valsalva. Furthermore, it was an acquired fistula due to vegetative endocarditis with invasion into the RCA and atrial septum, forming a communication with the RA (21). In our case, since there was no evidence of vegetative endocarditis, such as vegetative lesions, erosive lesions, abscess, or greater than trivial valvular insufficiency (22), the aorta-RA tunnel was considered a congenital anomaly; this is the first case in a dog in which the tunnel originates from the right coronary sinus of Valsalva and enters the RA.

Comparing our case with human medicine, we found some commonalities and differences. In both our case and aorta-RA tunnel in human medicine, the right-sided continuous murmur was confirmed (10–15). As for the symptoms, the dog, in our case, was asymptomatic. Similarly, in many cases in human medicine, the patients were asymptomatic (10–12, 14). Most patients had no significant cardiac remodeling, and only a few cases showed RV hypertrophy (14). Aorta-RA tunnel may be asymptomatic (10–12, 14), or it may present with shortness of breath, palpitations, or recurrent respiratory tract infections (13–15). Since blood generally flows from the left heart with relatively high pressure to the right heart with relatively low pressure in the presence of cardiac shunts (23), the aorta-RA tunnel behaves like a left-to-right shunt at the atrial level (15). Atrial level shunts often result in RV volume overload, and increased RV systolic pressure can result in significant RV hypertrophy. However, although pulmonary blood flow is increased, RV and pulmonary artery pressures rarely show significant elevation (24). Furthermore, the shunt size, which measures the excess volume of pulmonary blood flow, results in almost all of the significant clinical features directly or indirectly (24). Considering that a Qp/Qs ratio of 1.5 or less is considered a small shunt in general (25), a small shunt in our case (Qp/Qs ratio of 1.3) may have contributed to it being asymptomatic with mild LV volume overload. In our case, mild LV volume overload and decreased systolic function were thought to be caused by increased pulmonary flow due to the aorta-RA tunnel which behaves like a left-to-right shunt. Moreover, our case differs from previous cases in human medicine in that there were multiple fistulas.

A blood vessel observed in the interventricular septum on echocardiography was considered as part of the RCA or septal branch of the LCA. In the past, coronary arteries supplying the interventricular septum were known only as the septal branch of the LCA (26–30); however, a significant RCA supply to the canine interventricular septum was revealed in 1977 (31). The possibility of a coronary cameral fistula was considered due to a communication between the blood vessel within the interventricular septum and the RV. And the mosaic pattern observed between the aorta and pulmonary artery was considered the possibility of the aortopulmonary window or a supracristal ventricular septal defect (9, 16). However, it was considered that the size of the defect would be small because there was no clearly identified defect in the B-mode images. In addition, the presence of intrapulmonary arteriovenous anastomoses which was not clearly identified in CT, was demonstrated with saline contrast echocardiography. Therefore, it was considered subclinical microvascular pulmonary arteriovenous anastomoses (17, 32).

In our study, the aorta-RA tunnel was diagnosed using two-dimensional echocardiography and CT. It was confirmed by echocardiography and CT was helpful complementary diagnostic tool, especially in our case which was extracardiac shunt. Echocardiography may not be sufficient for assessment of extracardiac structures, CT was useful for evaluating extracardiac systemic and pulmonary arterial and venous structures. Two-dimensional reformation and three-dimensional reconstruction provided accurate depiction of morphologic structures of the aorta-RA tunnel. On the other hand, there were several disadvantages of CT compared to echocardiography. CT required general anesthesia, use of an iodinated intravenous contrast agent and was associated with radiation.

Our study has some limitations. First, aortography and coronary angiography were not performed. In most cases in human medicine, aortography and coronary angiography are performed to identify the aorta-RA tunnel and courses of the tunnel and coronary arteries. Therefore, in our case, it was difficult to clarify the course of the coronary arteries. However, CT examination showed that the morphologic structure of the main shunt (aorta-RA tunnel) could be more clearly visualized. Second, no treatment was undertaken in our patient due to the owner’s decision. In human medicine, because the patency of the communication can result in the enlargement of both ventricles, bacterial endocarditis, aneurysm formation, spontaneous rupture, and increased surgical mortality with age, the closure of an aorta-RA tunnel is recommended (11–13, 15). However, the need for surgical treatment in asymptomatic patients remains controversial (11).

To the best of our knowledge, this is the first report to describe an aorta-RA tunnel in a dog. In conclusion, if a right-sided continuous murmur is heard or shunt flow originating from the aortic root is identified in dogs, an aorta-RA tunnel should be included in the clinical differential diagnosis list.

## Data availability statement

The original contributions presented in the study are included in the article/supplementary material, further inquiries can be directed to the corresponding author.

## Author contributions

GK and HY: conception and design and drafting the article. GK, H-GJ, and HY: acquisition of data. GK, YJ, H-GJ, TL, KL, and HY: analysis and interpretation of data and revising article for intellectual content. All authors contributed to the article and approved the submitted version.

## Funding

This research was supported by Basic Science Research Program through the National Research Foundation of Korea (NRF) funded by the Ministry of Education (2019R1A6A1A03033084).

## Conflict of interest

The authors declare that the research was conducted in the absence of any commercial or financial relationships that could be construed as a potential conflict of interest.

## Publisher’s note

All claims expressed in this article are solely those of the authors and do not necessarily represent those of their affiliated organizations, or those of the publisher, the editors and the reviewers. Any product that may be evaluated in this article, or claim that may be made by its manufacturer, is not guaranteed or endorsed by the publisher.

## Glossary

Ao: Aorta
CT: Computed tomography
EDVI: End-diastolic volume index
ESVI: End-systolic volume index
EF: Ejection fraction
FOV: Field of view
FS: Fractional shortening
IVRT: Isovolumic relaxation time
LA: Left atrium
LA/Ao: Left atrial to aortic diameter ratio
LC: Left coronary cusp
LCA: Left coronary artery
LV: Left ventricle
LVIDDN: Left ventricular end-diastolic internal diameter corrected for body weight
LVIDSN: Left ventricular end-systolic internal diameter corrected for body weight
LVOT: Left ventricular outflow tract
MPA: Main pulmonary artery
NC: Non-coronary cusp
Qp/Qs: Pulmonary to systemic flow ratio
RA: Right atrium
RC: Right coronary cusp
RCA: Right coronary artery
RV: Right ventricle
TV: Tricuspid valve
